# Digital vs. Freehand Anterior Single-Tooth Implant Restoration

**DOI:** 10.1155/2020/4012127

**Published:** 2020-10-22

**Authors:** D. Baldi, J. Colombo, F. Motta, F. M. Motta, A. Zillio, N. Scotti

**Affiliations:** ^1^Department of Surgical Sciences (DISC), Division of Prosthetic Dentistry, University of Genoa, Genoa, Italy; ^2^Department of Surgical Sciences, University of Turin, Turin, Italy

## Abstract

Replacing a single tooth in the anterior maxilla is one of the greatest challenges in dentistry. Both functional and aesthetic results are to be strictly pursued. Planning and executing such a case through a totally digital methodology eventually guarantee many advantages, above all patient's operative and postoperative comfort. To ascertain this, a BOP analysis was performed which allowed us to evaluate soft tissues health, and more; crestal bone resorption was measured to evaluate hard tissues stability. This assumption was studied through four cases in which patients were alternatively treated with analogic and digital techniques. Four homogeneous patients were recruited. They all needed to extract one of the upper incisors, due to different clinical reasons, and then to replace it with an implant. Each patient was treated with an immediate postextractive implant which was immediately loaded, and finally, analogical and digital techniques were compared. All patients underwent a preoperative CBCT examination. After surgery, patients were checked by the surgeon after 10 days and one month to evaluate the progress of healing and to exclude any prosthetic problem. At 6 months (T1), one year (T2), and three years (T3), intraoral x-rays were performed using customized centring devices, according to the parallel beam technique. All data have been collected in a table and statistically processed; mean and standard deviation were measured. All patients entered an oral hygiene program with six months recall. Dental hygienist checked the BOP at T1, T2, and T3. At every step, similar levels of BOP were recorded. About interproximal bone loss, all patients showed an initial moderate loss (between T1 and T2), followed by stable values between T2 and T3. Despite the important limitations of a study with few cases, these results show a similar outcome comparing digital and analogical methods.

## 1. Introduction

Oral health seems to be of paramount importance for individuals [1, 2]. Replacing an anterior tooth in the maxillary area is one of the greatest rehabilitative challenges in dentistry [3–5]. In oral therapy, functional and aesthetic aims cannot be separated [6]. The great challenge for the dentist lies in perfectly blending surgery with the prosthetics in order to get an overall satisfactory result [7–9]. Furthermore, therapy must guarantee stability over time. The first step is a good surgical planning. A complete analysis of the implant site, a correct choice of the fixture [10–14], and its positioning are fundamental steps. Eventually, the choice of both temporary and definitive prosthesis accomplishes the path so that an optimal result is achievable. In recent years, new digital instruments would contribute to optimize all these steps, making the path predictable at all. Moreover, guided surgery let us achieve decidedly higher levels of precision compared to traditional techniques [15–19]. Furthermore, using an optical impression also optimize prosthetic steps [20–22]. Transition from a completely analogical to a completely digital execution makes these complex therapies more predictable, because these procedures become less operator depending. In particular, planning surgery through a dedicated software allows to pursue all parameters leading to long-term success. Differently, even in the hands of very experienced operators, traditional surgery can expose to inevitable errors due to anatomical and human factors. Restoration guides our digital planning in which every aspect can be optimize, including the abutment shape, the profile of the crown, and choice of materials [23, 24].

Furthermore alternative surgical methods, like Piezosurgery [25–28], would add patient's comfort. Various methods are analyzed starting from the assumption that they all biologically valid. For this reason, a BOP analysis was also performed to evaluate the health of soft tissues and interproximal crestal bone resorption to evaluate the stability of hard tissues [29]. The aim of the article is to analyze them with the help of four cases in which analog and digital modes alternate.

## 2. Materials and Methods

**Figure 1:**
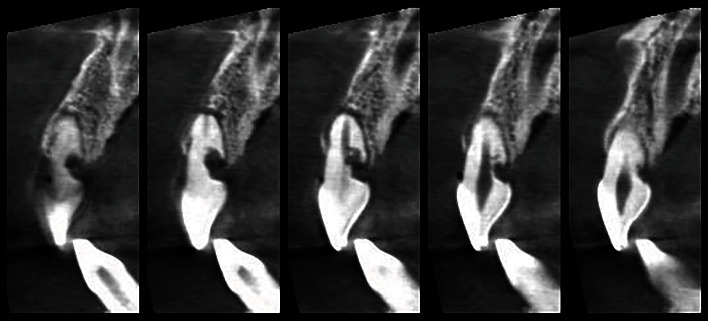
Preoperatory CBCT of the patient.

**Figure 2:**
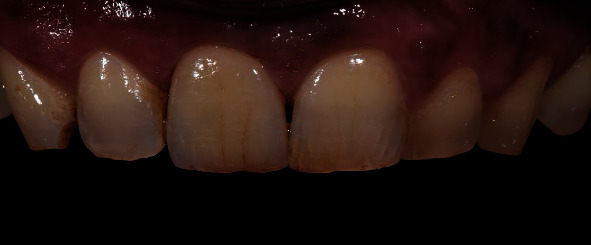
Final situation of patient 1.

**Figure 3:**
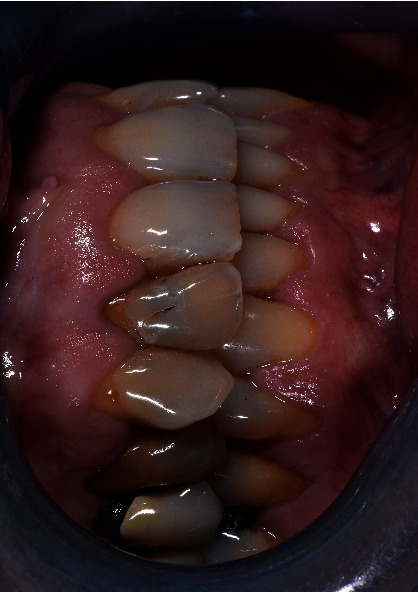
Preoperatory situation of patient 4.

**Figure 4:**
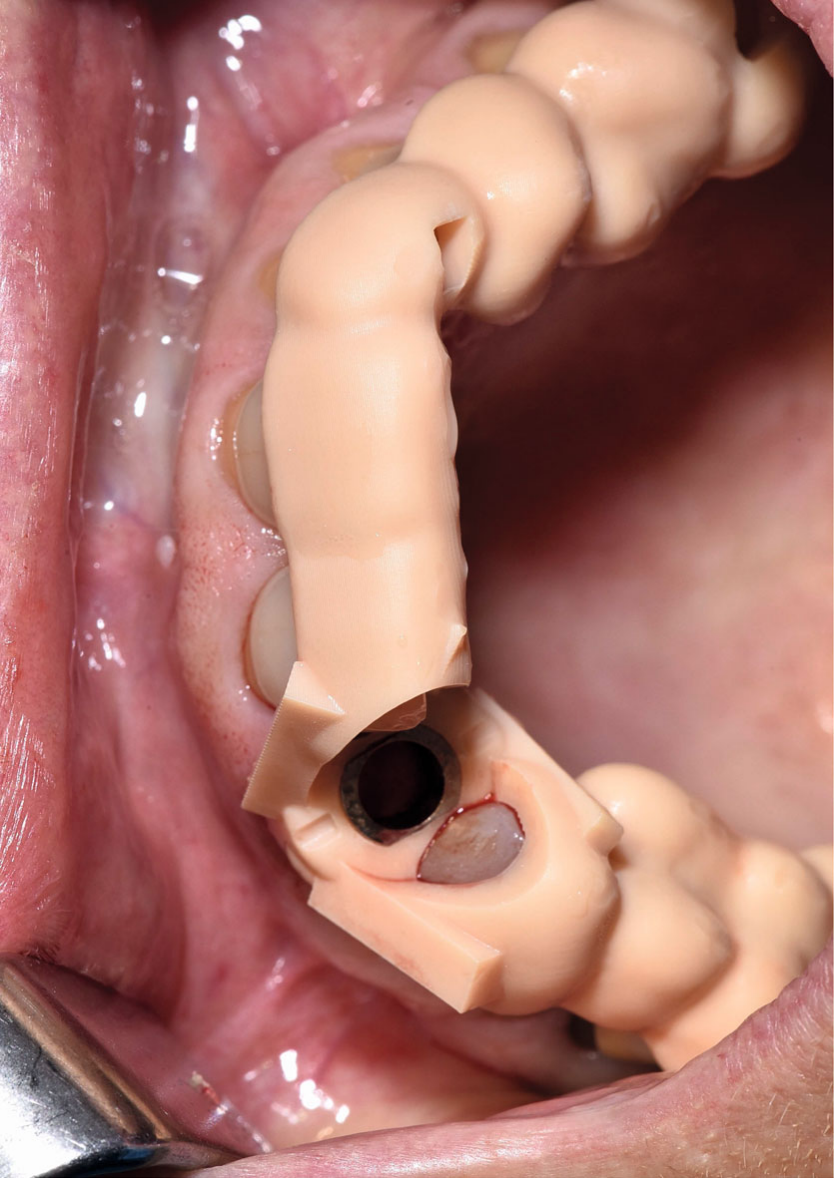
Surgical guide of patient 4.

**Figure 5:**
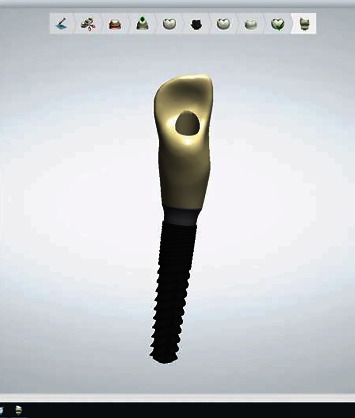
Provisional crown digital project of patient 4.

**Figure 6:**
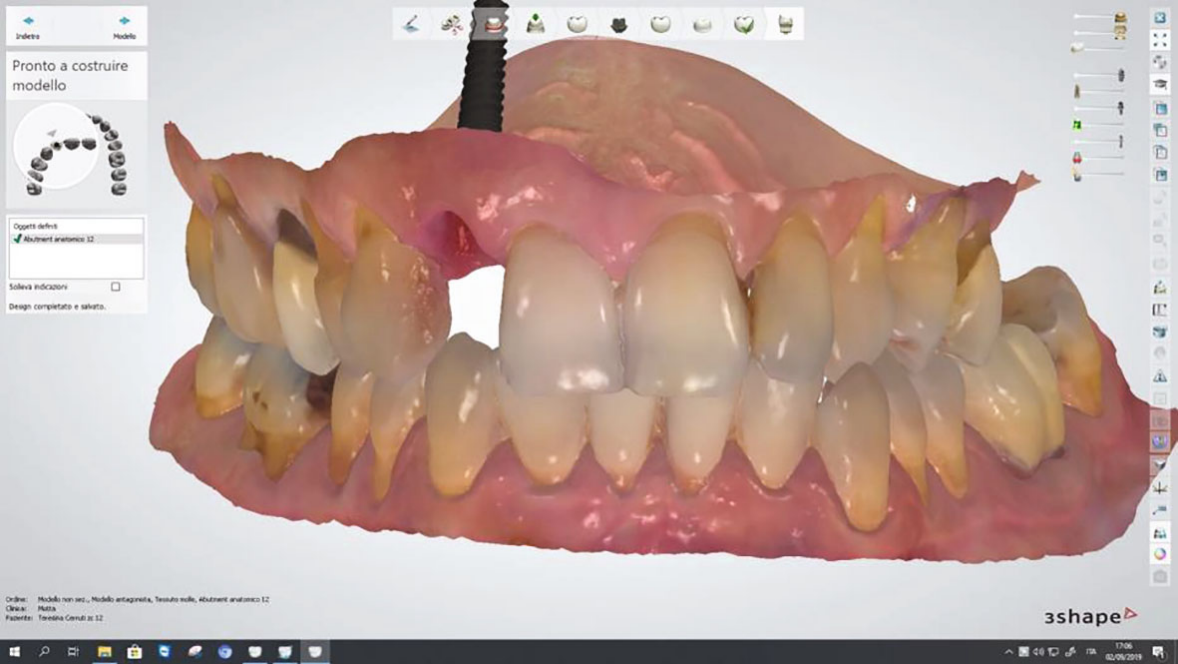
Definitive crown digital impression of patient 4.

**Figure 7:**
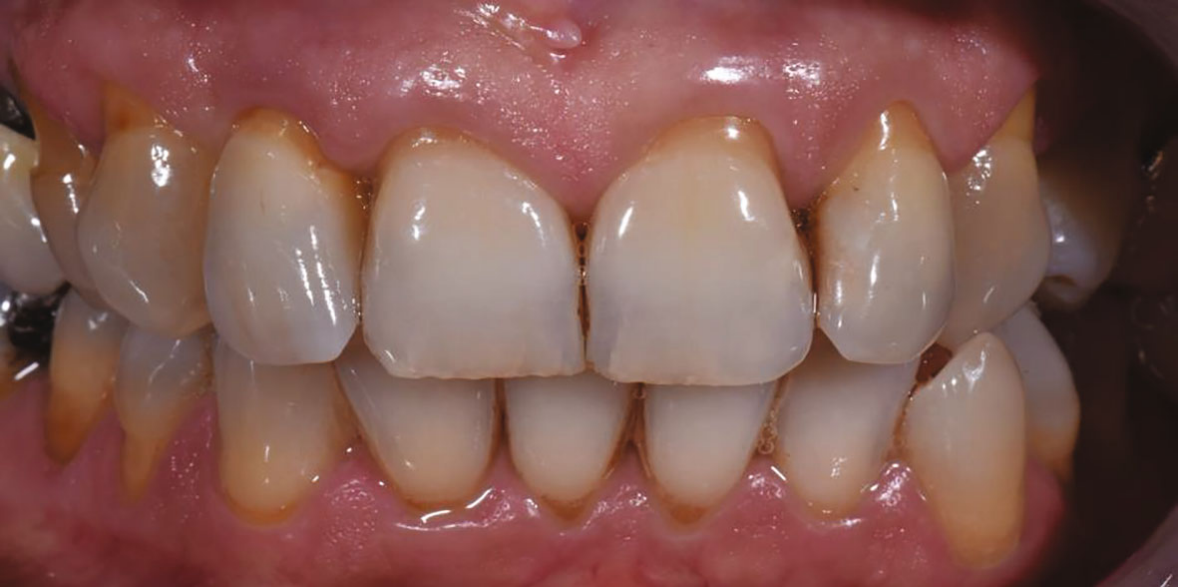
Final restoration *in situ* (patient 4).

Four patients have been recruited for the study, everyone being required for the extraction of an upper incisor and a related implant rehabilitation. All patients were in good health, and none presented contraindications to implant surgery. Informed consent was collected for all patients, and all possible methods of treatment were proposed. This prospective protocol has been led in accordance with the Good Clinical Practice Guidelines (GCPs) and following the recommendations of the Declaration of Helsinki as revised in Fortaleza (2013) for investigations into human subjects. Patient 1 underwent the extraction of tooth 1.1 because of an external resorption in the palatine area (Figure 1). The implant was inserted freehand immediate postextraction mode (Shelta 3.8 X 11.5, Sweden & Martina, Due Carrare, Italy), and then, it was immediately loaded with a temporary resin crown built after a traditional impression. At 6 months, a definitive screwed ceramic zirconia crown was made still after taking a traditional polyether impression (Figure 2). Patient 2 underwent 1.1 extraction caused by a root fracture. A freehand postextraction implant was inserted (Nobel Active 3.5 X 15, Nobel Biocare, Zurich, Switzerland). The implant was immediately loaded with a temporary crown made by taking a traditional impression. At 6 months, a definitive ceramic zirconia crown was built. In patient 3 was placed in an immediate postextractive implant (Nobel Active 3.5 X 13, Nobel Biocare, Zurich, Switzerland) in seat 22 for palatine destructive caries. Implant was placed with guided surgery (Implant Studio, 3Shape, Copenhagen, Denmark). Optical impression for temporary (one-time one abutment) and final crown (zirconia-ceramic) was cemented directly from the immediate abutment file without performing a new impression (only position impression of the zirconia coping to detect soft tissues after healing). In patient 4 was placed in an immediate postextractive implant in site 12 for a coronoradicular fracture (Nobel Active 3.5 X 13, Nobel Biocare, Zurich, Switzerland) (Figure 3). Implant was positioned with guided surgery (Implant Studio, 3Shape, Copenhagen, Denmark) and loaded with an immediate provisional made before surgery and already luted to the abutment (specifying the planned position) (Figures 4 and 5). Final optical impression was taken to build a screwed zirconia-ceramic crown (Figures 6 and 7). All implants were inserted in postextractive mode with a 1.5 mm subcrestal position, to compensate a predictable bone resorption due to tooth extraction [30]. Immediate loading with screwed temporaries was performed in all patients. Every patient was checked at 10 days and one month by the surgeon to evaluate the progress of healing and to look at any prosthetic problems. All patients underwent a preoperative CBCT examination. At 6 months (T1), 1 year (T2), and 3 years (T3), plates with Rx-customized centring devices were used according to the parallel beam technique, and with these, a radiologist measured the peri-implant bone loss in relation to mesial and distal peaks of the adjacent teeth. All data have been inserted in a table and have been subjected to a statistical investigation with measurement of mean and standard deviation. The patients entered into a tight professional hygiene program with 6-month calls, which were preferably performed with the use of powders [31]. At 6 months (T1), 1 year (T2), and 3 years (T3) of recalls, a dental hygienist performed the BOP (bleeding on probing) measurement, pinning the result on the patient's medical record. The bleeding sites have been expressed as a percentage of the total of the surveyed sites (6 per implant). Data were entered into two tables, and the mean and standard deviation were calculated.

## 3. Results

The BOP measurements have been inserted in a table (Table 1). The observed data showed almost the same level of gingival inflammation in all 4 cases, at T1, T2, and T3 after the insertion of the final manufact.

The loss of interproximal bone with respect to the peak of the adjacent teeth was inserted in a table (Table 2).

In all 4 cases, the loss of interproximal bone, after a more sensible loss between T1 and T2, has stabilized on almost constant values between T2 and T3. The average of T1 bone levels was 0.0125 mm (standard deviation + −0.15 mm). The T2 mean was 1.03 mm (standard deviation + −0.12 mm). The mean at T3 was 1.3 mm (standard deviation + −0.09 mm).

No patient at 3 years old dropped out the recall and control sessions.

## 4. Discussion

Analyzing the BOP data, it has been observed that all patients showed an excellent level of oral hygiene with a very low inflammatory index. Right after surgery, all patients underwent oral hygiene sessions and strict home instructions. It could be possible that the presence of an important rehabilitation in the aesthetic area may increase patient's compliance about hygienic maintenance.

Interproximal bone loss data reflected the trend of bone resorption expected from the literature data [32]. The most important resorption has been occurred between T1 and T2, therefore within a year since an implant insertion.

As suggested by Schwartz-Arad et al. [31], a natural contraction of the alveolus has been compensated by a the subcrestal insertion of about 1.5 mm of the implant in immediate postextractive mode, in order to obtain a final iuxtaosseous positioning of the head of the implant at T1.

Between T2 and T3, the bone rearrangement is extremely limited, according to Van Steenberghe [33].

From a biological point of view, using a completely analogical or a completely digital technique does not seem to determine differences. Instead, it is very interesting to focus on the predictability of the intervention. According to several authors, the three-dimensional position of the implant is one of the most important factors for the long-term stability of the peri-implant tissues [34, 35]. In the analogical insertion mode, success is strongly delegated to the surgeon's manual ability. In particular, some parameters such as the axis and the insertion depth of the implant require precise capabilities that are not easily reproducible. On the opposite, the choice of guided surgery allows you to completely plan the intervention. Even with the intrinsic limits of this method, for example the precision of CBCT exams or the precision of the surgical masks, there is no doubt that the operations can be performed with a clearly superior reproducibility of some parameters [30, 36]. Choosing a digital prosthetic flow allows more advantages. For example, in immediate loading, the use of a definitive abutment right after surgery, applying the concept of “one time, one abutment” proposed by Degidi et al. [37, 38]. Patients' comfort is also very important [39], because they must not undergo a traditional intraoperative impression. In fact, the clinician has both the possibility of already getting the temporary crown ready before surgery and the possibility of performing a scan of the implant after positioning it and getting the temporary in a very short time. A digital flow allows the clinician greater rigor in the surgical field and flexibility in the prosthetic field, with the possibility of planning the prosthesis since the preoperative setting and even of modifying the project with extremely simple steps. Obviously, the transition from analogical to digital techniques requires an important learning curve, which, however, once addressed allows a significant simplification of any clinical procedures.

## 5. Conclusions

Despite limitations of a study with few cases, results show a substantial overlap between analogical and digital implant-prosthetic techniques about peri-implant tissues health.

The prosthetic workflow also offers several advantages related to ergonomics and comfort; in addition, digital techniques would seem guarantee greater stability of peri-implant tissues in the long term.

Monitoring patients over time and designing new clinical trials with a larger sample are needed to confirm these results.

## Figures and Tables

**Table 1:** BOP index on implant site at T1, T2, and T3.

	T1 (6 months)	T2 (1 year)	T3 (3 years)
Case 1	0	0	0
Case 2	0	16.6	0
Case 3	16.6	16.6	16.6
Case 4	0	0	16.6

**Table 2:** Interproximal bone loss from T1 to T3.

	T1 medial	T1 distal	T2 medial	T2 distal	T3 medial	T3 distal
Case 1	0.1	0.2	1	1.1	1.2	1.2
Case 2	0	-0.2	1.1	1.1	1.3	1.4
Case 3	-0.2	-0.1	0.9	0.8	1.2	1.3
Case 4	0	0.1	1.1	1.1	1.4	1.4

## Data Availability

All data of the present article are available on request by contacting corresponding author.
